# Metformin combats high glucose-induced damage to the osteogenic differentiation of human periodontal ligament stem cells via inhibition of the NPR3-mediated MAPK pathway

**DOI:** 10.1186/s13287-022-02992-z

**Published:** 2022-07-15

**Authors:** Yi-Lin Zhang, Fen Liu, Zhi-Bang Li, Xiao-Tao He, Xuan Li, Rui-Xin Wu, Hai-Hua Sun, Shao-Hua Ge, Fa-Ming Chen, Ying An

**Affiliations:** 1 State Key Laboratory of Military Stomatology and National Clinical Research Center for Oral Diseases and Shaanxi Engineering Research Center for Dental Materials and Advanced Manufacture, Department of Periodontology, School of Stomatology, Fourth Military Medical University, Xi’an, 710032 People’s Republic of China; 2 Department of Stomatology Northwest Women’s and Children’s Hospital, Xi’an, Shaanxi People’s Republic of China; 3 Department of Periodontology, School and Hospital of Stomatology, Cheeloo College of Medicine, Shandong University and Shandong Key Laboratory of Oral Tissue Regeneration and Shandong Engineering Laboratory for Dental Materials and Oral Tissue Regeneration, Jinan, Shandong China

**Keywords:** High glucose, Metformin, Osteogenic differentiation, NPR3, MAPK pathway, Periodontal tissue regeneration

## Abstract

**Background:**

High glucose-induced damage to the osteogenic differentiation of human periodontal ligament stem cells (PDLSCs) has long been a challenge to periodontal regeneration for diabetic individuals. Metformin is an anti-hyperglycemic drug that exhibits abundant biological activities associated with cell metabolism and downstream tissue regeneration. However, how metformin combats damage to PDLSC osteogenic differentiation under high glucose and the underlying mechanisms remain unknown.

**Methods:**

Osteogenic differentiation of PDLSCs was assessed by alkaline phosphatase (ALP) staining, ALP activity, Alizarin Red staining and quantitative assay, quantitative real-time polymerase chain reaction (qRT-PCR) and Western blot analysis. RNA-seq analysis was performed to screen target genes of metformin, and the effects of target genes were confirmed using lentivirus transfection. Western blot analysis was also used to detect the protein level of underlying signaling pathways.

**Results:**

We found that osteogenic differentiation of PDLSCs under high glucose was decreased, and metformin addition enhanced this capacity of differentiation. Furthermore, the results of RNA-seq analysis showed that natriuretic peptide receptor 3 (NPR3) was upregulated in PDLSCs under high glucose and downregulated after metformin addition. When the underlying pathways involved were investigated, we found that upregulation of NPR3 can compromise the metformin-enhanced PDLSC osteogenic differentiation and activate the MAPK pathway (especially the p38 MAPK and Erk1/2 pathway), and that inhibition of the NPR3-mediated p38 MAPK or Erk1/2 pathway enhanced the osteogenic differentiation of PDLSCs under high glucose.

**Conclusions:**

The present study suggests that metformin may enhance the osteogenic differentiation of PDLSCs under high glucose via downregulation of NPR3 and inhibition of its downstream MAPK pathway. This is the first report identifying the involvement of NPR3-mediated MAPK pathway in the metformin-enhanced osteogenic differentiation, indicating that NPR3 antagonists, such as metformin, may be feasible therapeutics for periodontal tissue regeneration in diabetic individuals.

**Supplementary Information:**

The online version contains supplementary material available at 10.1186/s13287-022-02992-z.

## Background

Periodontal disease constitutes a group of chronic inflammatory processes characterized by destruction of supporting structures of teeth [1]. This disease has been linked to more than 50 systemic diseases, mainly including cardiovascular disease and diabetes mellitus [2, 3]. In particular, periodontal disease has been recognized as one of the most prevalent complications of diabetes mellitus [4]. Compared to individuals without diabetes, patients with diabetes are more susceptible to periodontal disease, and the damage caused by periodontal disease could possibly be exacerbated in these patients [5, 6]. In addition, evidence has suggested that the hyperglycemic environment in diabetic patients leads to delayed periodontal wound healing and impaired tissue regeneration [7–9]. Therefore, it is essential to enhance our knowledge about the impact of diabetes on periodontal disease and indeed to identify new agents that can promote periodontal regeneration in diabetic individuals.

Mesenchymal stem cells (MSCs) are multipotent somatic stem cells that can be isolated from a variety of human tissues, including bone marrow, adipose tissue and periodontal ligament (PDL) [10]. These cells have become promising candidates for tissue engineering and regenerative medicine [11]. Periodontal ligament stem cells (PDLSCs) are important sources of MSCs that can be isolated from PDL tissues without significant safety or ethical concerns [12]. Moreover, PDLSCs isolated from periodontal ligament tissues can still survive, proliferate and form periodontal ligament-like tissues after in vivo transplanted [13, 14]. With their advantage of multilineage differentiation, PDLSCs are becoming an attractive option for regeneration of destroyed periodontal tissues [15]. However, high glucose-induced damage to PDLSCs presents a major challenge to their applications in diabetic individuals [16]. Accumulating evidence indicates that PDLSCs incubated in high glucose exhibit impaired stem cell performance, including reduced proliferation and multilineage differentiation potentials, which would compromise the therapeutic effect of these cells [17, 18]. Therefore, enhancing the stemness of PDLSCs under high glucose is important for periodontal tissue regeneration in diabetic individuals.

Metformin is a first-line drug extensively used for the treatment of diabetes [19]. In addition to its hypoglycemic effect, metformin also possesses anti-aging, anti-inflammatory and immunoregulatory activities, indicating that this drug has great potential for the treatment of other disorders [20–22]. In recent years, the number of studies focusing on the role of metformin in the treatment of MSCs has been increasing. This drug has been reported to improve cellular proliferation and multilineage differentiation potentials in vitro and to enhance the survival of stem cells in vivo [23–25]. In addition, several studies have also demonstrated that metformin treatment can rescue the reduced differentiation potential of rat adipose-derived stem cells under high glucose [26]. However, how metformin combats damage to PDLSC osteogenic potential under high glucose and the underlying mechanisms remain unknown.

In this study, we hypothesize that metformin may confer a protective effect on the osteogenic differentiation of PDLSCs under high glucose. To test our hypothesis, we first measured the osteogenic differentiation of PDLSCs under normal or high glucose. Next, metformin was added to the culture medium to investigate its biological function in PDLSC osteogenic differentiation under high glucose. Furthermore, we screened and identified the potential genes and signaling pathways involved in metformin-mediated PDLSC osteogenic differentiation. Our findings will provide new insight into the regulation of cellular osteogenic differentiation under high glucose, which is beneficial for optimizing current or developing new regenerative paradigms for diabetic individuals.

## Methods

### Isolation and culture of PDLSCs

Human PDL tissue samples were obtained from third molars or teeth extracted for orthodontic reason. Written informed consent was provided by all donors (*n* = 5, 3 males and 2 females, aged 18–24 years), and the subsequent studies were approved by the Ethics Committee of the School of Stomatology, Fourth Military Medical University, Xi’an, Shaanxi, China. In summary, freshly extracted teeth were washed using cold phosphate-buffered saline (PBS; Corning, New York, USA), and gingival tissue was excluded. PDL tissues were scraped from the middle third of the root surface and cut into small pieces (approximately 1 mm^3^). Next, the periodontal samples were digested by type I collagenase (Sigma-Aldrich, St. Louis, USA) for 45 min in dark. After digestion, the PDL tissues were resuspended in complete Dulbecco’s modified Eagle’s medium (DMEM; Gibco, New York, USA) supplemented with 10% (v/v) fetal bovine serum (FBS; Sijiqing, Hangzhou, China), 1% penicillin (Invitrogen, Carlsbad, CA, USA) and streptomycin (Invitrogen). The PDL tissues were finally transferred to a 6-well culture plate (Invitrogen) and cultured at 37 °C in a humidified atmosphere of 95% air and 5% CO_2_. The medium was exchanged every 2 days. These cells were recognized as P0 cells. When these P0 cells reached 80–90% confluence, they were digested and seeded in 100-mm-diameter culture dishes (Invitrogen). The cells cultured in the dishes were recognized as P1 cells. Then, these cells were cultured and passaged when they reached 80–90% confluence until P4. To eliminate the individual differences in cells from different donors, P4 cells from the same donors were labeled and cryopreserved in liquid nitrogen using CELLSAVING (New Cell & Molecular Biotech, Suzhou, China) and thawed at the same time for cellular identification and further experiments.

### Flow cytometry analysis

Flow cytometry analysis was conducted to identify the immunophenotypes of PDLSCs. In brief, P4 cells from the five donors were collected and then transferred into sterile Eppendorf tubes (Invitrogen) at 5 × 10^5^ cells per tube. The cells were incubated with monoclonal antibodies against human CD73, CD90, CD105, CD146, CD11b, CD19, CD31, CD34, CD45 and HLA-DR (all from eBioscience, San Diego, USA) according to the ISCT definition of MSCs and previous studies [27–29]. Cells without antibodies served as the blank control. After labeling for 1 h in dark, the PDLSCs were washed and resuspended with PBS. These procedures were performed on ice. The immunophenotypes of these cells were assessed with a Beckman Coulter Epics AL cytometer (Beckman Counter, Fullerton, USA).

### Colony-forming unit (CFU) assay

To assess colony-forming efficiency of PDLSCs, P4 cells were seeded in 100-mm-diameter culture dishes at 1 × 10^3^ cells/dish. The medium was exchanged every 2 days. After 14 days of cultivation, the cells were fixed using 4% paraformaldehyde (Invitrogen) for 30 min and then stained with 0.1% toluidine blue (Sigma-Aldrich) for 20 min. Cell colonies were photographed with an inverted microscope (Olympus Optical, Tokyo, Japan). Cell aggregates containing more than 50 cells were recognized as colonies.

### Cell Counting Kit-8 (CCK-8) assay

Proliferation ability of PDLSCs was measured with Cell Counting Kit-8 assay. P4 cells were seeded in 96-well culture plates (Invitrogen) at a density of 1 × 10^3^. After 24 h of cell adhesion, 200 μL medium with 20 μL CCK-8 reagent (Invitrogen) was added to the test wells. The plate was then incubated at 37 °C for 2 h. After incubation, absorbance at 450 nm was detected with a microplate reader (TECAN, Männedorf, Switzerland) to measure the proliferation capacity of PDLSCs. During the 8-day culture, all procedures mentioned were performed at proscribed time points every day.

### Osteogenic differentiation assay

For osteogenic induction, P4 cells were seeded in 6-well plates at a density of 1 × 10^5^ until 70–80% confluence. Cells were then cultured with osteo-inductive medium: complete DMEM or high glucose (25 mmol/L) DMEM (Gibco) supplemented with 10% (v/v) FBS, 1% penicillin and streptomycin, 50 μg/mL vitamin C (Sigma-Aldrich), 10 nM dexamethasone (Sigma-Aldrich) and 10 mM β-glycerophosphate (Sigma-Aldrich). The medium was exchanged every 2 days.

Alkaline phosphatase (ALP) staining was performed after osteogenic induction for 7 days. PDLSCs were stained using a BCIP/NBT ALP Color Development kit (Biotime, Shanghai, China) and then observed and photographed with an inverted microscope. ALP activity measurement was performed using an ALP assay kit (Nanjing Jiancheng Bioengineering Institute, Nanjing, China). Alizarin Red staining (Sigma-Aldrich) was conducted after osteogenic induction for 21 days. PDLSCs were fixed using 4% paraformaldehyde (Invitrogen) for 30 min. Calcium deposits were stained and then observed and photographed with an inverted microscope. The stained areas were then dissolved in 6% cetylpyridine (Sigma-Aldrich) for 15 min, and the absorbance at 560 nm was assessed using a microplate reader for quantitative assay.

### Adipogenic differentiation assay

For adipogenic induction, P4 cells were seeded in 6-well plates at a density of 1 × 10^5^ until 80–90% confluence. Cells were then cultured with adipo-inductive medium (Cyagen, Guangzhou, China). The medium was exchanged every 2 days.

Oil red O staining (Sigma-Aldrich) was performed after adipogenic induction for 21 days. PDLSCs were fixed using 4% paraformaldehyde (Invitrogen) for 30 min. Lipid droplets were stained and then observed and photographed with an inverted microscope. The stained areas were then dissolved in isopropanol (Sigma-Aldrich) for 15 min, and the absorbance at 560 nm was measured using a microplate reader for quantitative assay.

### Chondrogenic differentiation assay

For chondrogenic induction, P4 cells were collected in 15-mL centrifuge tubes (Invitrogen) at a density of 2 × 10^5^ cells/tube. The cells were washed with PBS and then resuspended in chondro-inductive medium (Cyagen). After 24 h, cell spheres were visible at the bottom of the tubes. The medium was exchanged every 2 days.

According to studies reported [30], Alcian blue staining was performed after chondrogenic induction for 21 days to measure the chondrogenic differentiation ability of PDLSCs.

### Selection of the optimal concentration of metformin

To select the optimal concentration of metformin (Sigma-Aldrich), the toxic and protective effects of metformin were assessed by CCK-8 assay. For toxic effect, PDLSCs were seeded in 96-well culture plates at a density of 5 × 10^3^ with different concentrations of metformin (0, 10, 100, 500 and 1000 µM). Cell viability was measured at 6, 12, 24, 48 and 72 h later. For protective effect, PDLSCs were seeded in 96-well culture plates at a density of 1 × 10^3^ with the aforementioned concentrations of metformin. Cell viability was measured daily during the 6-day culture period. Accordingly, concentrations of metformin that induced cell toxicity were excluded. Ultimately, a concentration that conferred the maximal protective effect was selected.

### RNA-seq analysis

RNA-seq analysis was performed to detect differentially expressed genes in PDLSCs cultured in different osteo-inductive mediums. In brief, total RNA was extracted using a TRIzol reagent kit (Invitrogen). RNA quality was assessed using an Agilent 2100 Bioanalyzer (Agilent Technologies, Palo Alto, USA) and verified using RNase-free agarose gel electrophoresis. Then, eukaryotic mRNA was enriched with Oligo (dT) beads, and total RNA was fragmented into short fragments using fragmentation buffer and reversely transcribed into cDNA with random primers. Second-strand cDNA was synthesized by DNA polymerase I, RNase H, dNTP and buffer. The cDNA fragments were purified with a QiaQuick PCR extraction kit (Qiagen, Venlo, The Netherlands), and then, the ends were repaired, and poly(A) was added. The fragments were ligated to Illumina sequencing adapters. Desired genes were selected and compared to determine changes in the expression levels of relevant genes. After purification, cDNA fragments were sequenced using an Illumina Novasqr6000 by Gene Denovo Biotechnology Co (Guangzhou, China).

### Lentivirus transfection

Lentivirus transfection was performed to stably upregulate the expression of natriuretic peptide receptor 3 (NPR3) in PDLSCs. In brief, PDLSCs were cultured in 6-well plates at a density of 1 × 10^5^ until 40 ~ 50% confluence. Then, the cells were transfected with NPR3-overexpression lentivirus (NM_001204375.2) in the presence of polybrene (GenePharma, Shanghai, China). Cells transfected with LV5 lentiviral vector were used as negative controls. The medium was exchanged 24 h after transfection. NPR3-overexpression lentivirus as well as LV5 lentiviral vector were packaged by GenePharma (Shanghai, China).

The transfection efficiency of the lentiviral vector was confirmed using phase contrast and fluorescence microscopy; and the efficiency of lentivirus-mediated upregulation of NPR3 expression was confirmed by qRT-PCR, Western blot analysis and immunofluorescence staining.

### Enzyme-linked immunosorbent assay (ELISA)

After 7 days of osteogenic induction, the culture medium was collected and centrifuged to remove the cells and debris. The concentrations of C-type natriuretic peptide (CNP) secreted into the cell culture supernates were detected with an ELISA kit (Rebiosci, Shanghai, China).

### Quantitative real-time polymerase chain reaction (qRT-PCR)

Quantitative real-time polymerase chain reaction (qRT-PCR) was conducted to measure mRNA expression levels following the manufacturer’s instructions. In brief, Total RNA was extracted from cells with TRIzol reagent (Invitrogen). Extracted total RNA was reverse-transcribed to cDNA using Evo M-MLV RT Premix (Takara, Shiga, Japan). Quantitative real-time PCR was performed using the SYBR Green Premix Pro Taq HS qPCR kit (Tli RNaseH Plus; TaKaRa), and the results were analyzed with a CFX96 Real-time RT-PCR system (Bio-Rad, Hercules, CA, USA). Glyceraldehyde 3-phosphate dehydrogenase (GAPDH) was used to normalize the expression levels. The expression of target genes was calculated using the 2^−ΔΔCT^ method. The primer sequences used for qRT-PCR are listed in Table 1.

**Table 1:** Sequences of gene-specific primers used in the present study

Gene ID	Genes	Forward sequence	Reverse sequence
632	*OCN*	CCCAGGCGCTACCTGTATCAA	GGTCAGCCAACTCGTCACAGTC
860	*RUNX2*	TGGTTACTGTCATGGCGGGTA	TCTCAGATCGTTGAACCTTGCTA
249	*ALP*	AACATCAGGGACATTGACGTG	GTATCTCGGTTTGAAGCTCTTCC
2597	*GAPDH*	GGAGTCCACTGGCGTCTTCA	GTCATGAGTCCTTCCACGATACC
60	*β-Actin*	CTGCTCATCCCACTAATGTC	CTTTATTAACTACCACCTGGTCCT
650	*BMP2*	ACCCGCTGTCTTCTAGCGT	TTTCAGGCCGAACATGCTGAG
4880	*CNP*	CTGACCGACCGACTCCAG	AAATCTCTCACCTCCGCCAG
374	*AREG*	GTGGTGCTGTCGCTCTTGATA	CCCCAGAAAATGGTTCACGCT
3488	*IGFBP5*	ACCTGAGATGAGACAGGAGTC	GTAGAATCCTTTGCGGTCACAA
8111	*GPR68*	TGTACCATCGACCATACCATCC	GGTAGCCGAAGTAGAGGGACA
6423	*SFRP2*	CTTAGAGGACAGCGGGGAAG	TCCAAGCATCTTGCCCTGAG
4883	*NPR3*	AGACTACGCCTTCTTCAACATTG	GCTTCAAAGTCGTGTTTGTCTCC

### Western blot analysis

Western blot analysis was performed to measure protein expression levels in PDLSCs. Briefly, the prepared cells were first lysed in RIPA lysis buffer (Biotime) supplemented with protease and phosphatase inhibitors (Biotime). A bicinchoninic acid (BCA) assay kit (Biotime) was then used to measure protein concentrations. Protein samples were separated by sodium dodecyl sulfate–polyacrylamide gel electrophoresis (SDS-PAGE; Biotime) and transferred to PVDF membranes (Millipore, Billerica, MA, USA). After blocking with 5% nonfat milk (Biotime) at room temperature for 2 h, the membranes were incubated with primary antibodies at 4 °C overnight. The membranes were then incubated with horseradish peroxidase (HRP)-conjugated secondary antibodies (1:2000; goat anti-rabbit IgG, CST, #7074; goat anti-mouse IgG, CST, #7076) at room temperature for 2 h. Subsequently, the blots were visualized using enhanced chemiluminescence substrate (ECL kit, Millipore), and protein bands were analyzed with ImageJ software. GAPDH was used as the housekeeping gene for internal normalization. The following primary bodies were used for Western blotting: GAPDH (1:3000; Affinity, AF7021), ALP (1:10000; Abcam, ab108337), RUNX2 (1:1000; CST, #12556), BMP2 (1:1000; Abcam, ab214821), OCN (1:1000; Santa Cruz; Sc-390877), NPR3 (1:1000; Abcam, ab177954), p38 MAPK (1:1000; CST, #9212), p-p38 MAPK (1:1000; CST, #4511), JNK (1:1000; CST, #9252), p-JNK (1:1000; CST, #4668), ERK1/2 (1:1000; CST, #9102) and p-ERK1/2 (1:1000; CST, #9101).

### Immunofluorescence staining

Immunofluorescence staining was performed to visualize NPR3 in PDLSCs. Briefly, cells were fixed with 4% paraformaldehyde for 30 min, followed by treatment with 0.5% Triton X-100 (Sigma-Aldrich) and 2% bovine serum albumin (BSA; Sigma-Aldrich). Then, the cells were incubated with primary antibodies against NPR3 (1:100; Abcam, ab97389) over night. The secondary antibodies for NPR3 were Alexa Fluor 488 AffiniPure donkey anti-rabbit IgG (1:100; Yeasen Biotech Company, Shanghai, China). Finally, the cell nuclei were counterstained with 4′,6-diamidino-2-phenylindole (DAPI; Abcam, ab104139), and the fluorescence images were acquired using an Olympus FV1000 confocal laser scanning microscope (CLSM; Olympus, Tokyo, Japan) and analyzed using FV10-ASW4.2 software.

### Statistical analysis

All data are presented as the mean ± standard deviation of at least three independent experiments. Statistical analysis was performed using GraphPad Prism 8 software. One-way analysis of variance followed by Tukey’s multiple comparisons tests, Sidak’s multiple comparisons tests or Dunnett's multiple comparisons was used for comparing more than two groups, and unpaired two-tailed Student’s t test was used for comparisons of two unpaired groups. Statistical significance was expressed as *p* < 0.05 (*), *p* < 0.01 (**) or *p* < 0.001 (***).

## Results

### Characterization of human PDLSCs

The results of CFU assay confirmed that the PDLSCs we obtained were plastic-adherent and can generate new colonies (Additional file 1: Fig. S1A). These cells continued to multiply, as suggested by the CCK-8 assay results (Additional file 1: Fig. S1B). The cells were also able to differentiate into multilineage cells when cultured in osteo-inductive, adipo-inductive and chondro-inductive medium, as evidenced by Alizarin red staining, Oil red O staining and Alcian blue staining, respectively (Additional file 1: Fig. S1C). In addition, flow cytometry analysis verified that these PDLSCs positively expressed CD73, CD90, CD105 and CD146, but negatively expressed CD11b, CD19, CD31, CD34, CD45 and HLA-DR (Additional file 1: Fig. S1D).

### High glucose impaired the osteogenic differentiation of PDLSCs

**Fig. 1:**
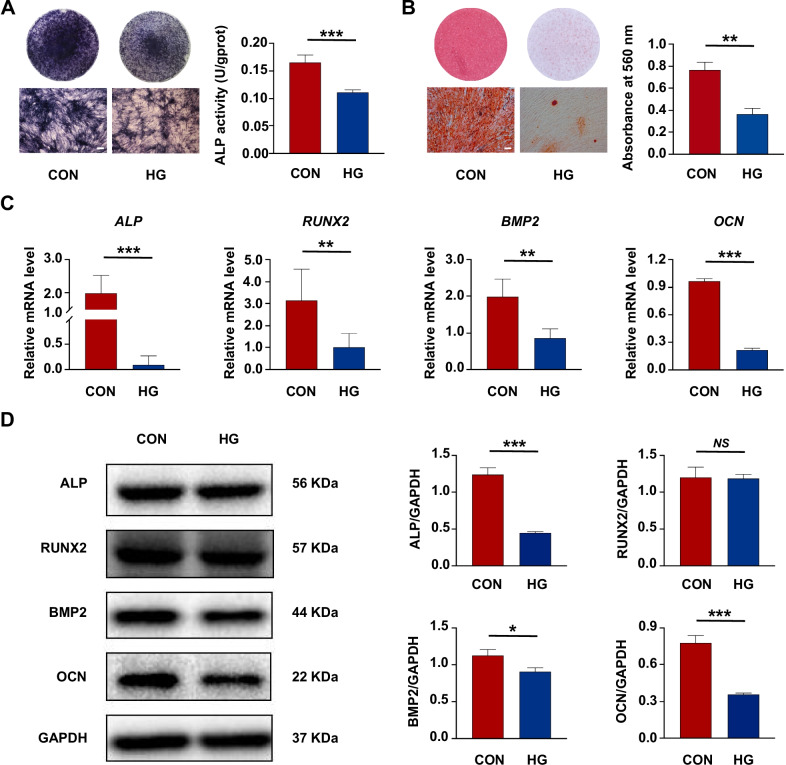
High glucose impaired the osteogenic differentiation of periodontal ligament stem cells (PDLSCs). **A** ALP staining and ALP activity assay of PDLSCs when they were cultured in normal (CON) or high glucose (HG) condition following a 7-day osteogenic induction (scale bar = 200 µm). **B** Alizarin Red staining and quantitative analysis of the stained calcium deposits formed by PDLSCs when they were cultured in CON or HG condition following a 21-day osteogenic induction (scale bar = 200 µm). **C** Expression of osteoblast differentiation-related genes (*ALP*, *RUNX2*, *BMP2* and *OCN*) in PDLSCs when they were cultured in CON or HG condition following a 14-day osteogenic induction (mRNA expression levels detected by qRT-PCR). **D** Expression of osteoblast differentiation-related proteins (ALP, RUNX2, BMP2 and OCN) in PDLSCs when they were cultured in CON or HG condition following a 14-day osteogenic induction (protein expression levels detected by Western blot analysis). The displayed bands were cropped from the corresponding original blots. Experiments for P4 cells from three different donors were repeated independently for at least 3 times, and data are presented as the means ± SD (*n* = 3). *p *value was based on *t* test. *p < 0.05, **p < 0.01, and ***p < 0.001 represent significant differences between the indicated columns, while *NS* represents no significant difference

The osteogenic differentiation of PDLSCs under normal or high glucose was measured respectively. We found that incubation of PDLSCs under high glucose led to fewer ALP staining-positive cells and lower cellular ALP activity (Fig. 1A). The results of Alizarin red staining further confirmed that less calcium deposits were formed by PDLSCs under high glucose compared to the control group (Fig. 1B). In addition, the results of qRT-PCR revealed that the osteoblast differentiation-related genes *ALP*, *RUNX2*, *BMP2* and *OCN* were downregulated in PDLSCs under high glucose (Fig. 1C). Similarly, the expression of osteoblast differentiation-related proteins ALP, BMP2 and OCN was also decreased in PDLSCs under high glucose, but no obvious change was observed in the protein level of RUNX2 (Fig. 1D).

### Metformin enhanced the osteogenic differentiation of PDLSCs under high glucose

To investigate the effect of metformin on the impaired PDLSC osteogenic differentiation under high glucose, metformin was added to the culture medium. We first selected the optimal concentration of metformin. During a 72-h incubation, cell viability was not significantly changed by different concentrations of metformin (0, 10, 100, 500 and 1000 µM) under high glucose (Additional file 2: Fig. S2A). In addition, PDLSCs treated with 100 µM metformin exhibited a higher proliferation rate than those in the other groups (Additional file 2: Fig. S2B). Hence, metformin at a concentration of 100 µM was selected for further studies.

**Fig. 2:**
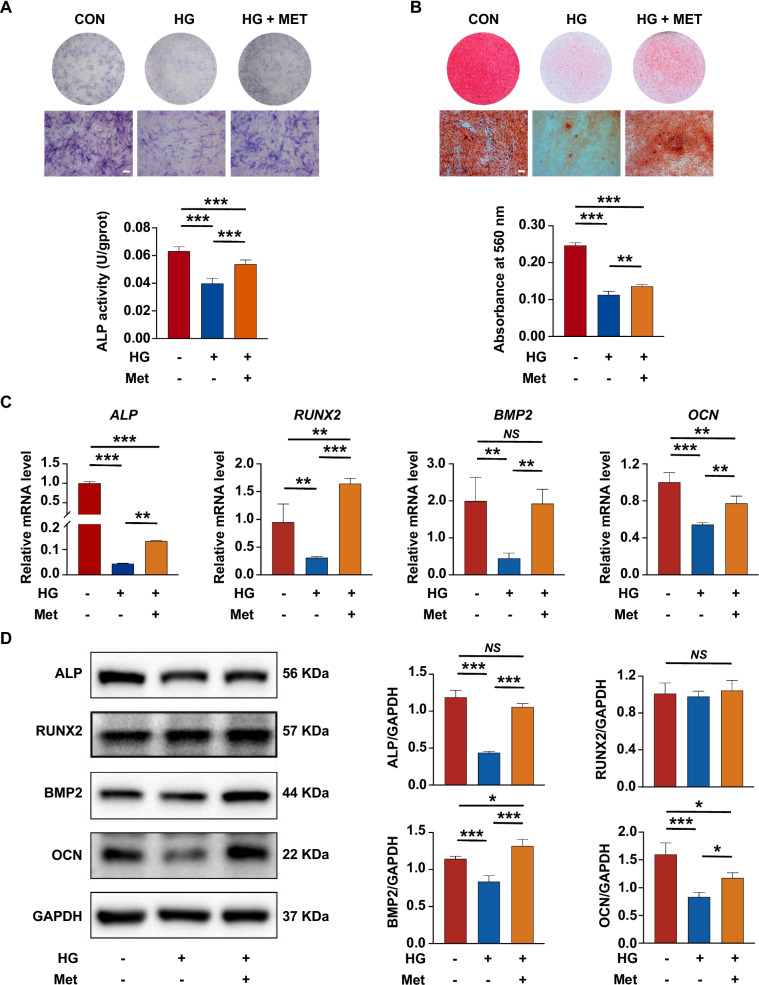
Metformin enhanced the osteogenic differentiation of PDLSCs under high glucose. **A** ALP staining and ALP activity assay of PDLSCs when they were cultured in normal (CON), high glucose (HG) or high glucose with metformin addition (HG + MET) following a 7-day osteogenic induction (scale bar = 200 µm). **B** Alizarin Red staining and quantitative analysis of the stained calcium deposits formed by PDLSCs when they were cultured in normal (CON), high glucose (HG) or high glucose with metformin addition (HG + MET) following a 21-day osteogenic induction (scale bar = 200 µm). **C** Expression of osteoblast differentiation-related genes (*ALP*, *RUNX2*, *BMP2* and *OCN*) in PDLSCs when they were cultured in normal, high glucose or high glucose with metformin addition following a 14-day osteogenic induction (mRNA expression levels detected by qRT-PCR). **D** Expression of osteoblast differentiation-related proteins (ALP, RUNX2, BMP2 and OCN) in PDLSCs when they were cultured in normal, high glucose or high glucose with metformin addition following a 14-day osteogenic induction (protein expression levels detected by Western blot analysis). The displayed bands were cropped from the corresponding original blots. HG, high glucose. Met, metformin. Experiments for P4 cells from three different donors were repeated independently for at least 3 times, and data are presented as the means ± SD (*n* = 3). *p *value was based on one-way analysis of variance (one-way ANOVA). *p < 0.05, **p < 0.01, and ***p < 0.001 represent significant differences between the indicated columns, while *NS* represents no significant difference

After metformin addition, the number of ALP staining-positive PDLSCs was increased, and ALP activity in cells was also enhanced (Fig. 2A). Consistently, the results of Alizarin red staining indicated that more calcium deposits were formed by PDLSCs with metformin addition than those without (Fig. 2B). Furthermore, the results of qRT-PCR revealed that after metformin addition, the gene levels of *ALP*, *RUNX2*, *BMP2* and *OCN* were significantly increased in PDLSCs under high glucose (Fig. 2C). The protein levels of ALP, BMP2 and OCN were also elevated after metformin addition, but no obvious change was observed in the protein level of RUNX2 (Fig. 2D).

### Screening and identification of osteogenesis-related genes mediating the metformin-enhanced osteogenic differentiation under high glucose

**Fig. 3:**
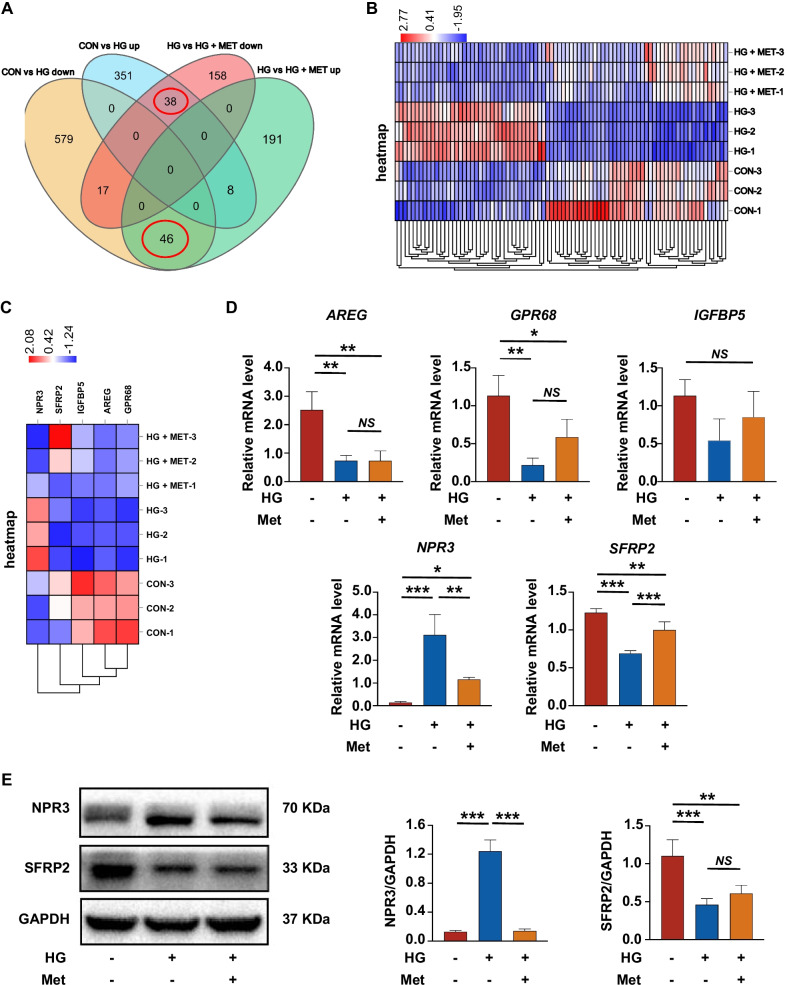
Screening and identification of osteogenesis-related genes mediating metformin-enhanced osteogenic differentiation under high glucose. **A** Venn diagram showing the number of differentially expressed genes in PDLSCs when they were cultured in normal (CON), high glucose (HG) or high glucose with metformin addition (HG + MET) following a 14-day osteogenic induction. **B** Heatmap showing all the differentially expressed genes in PDLSCs when they were cultured in normal (CON), high glucose (HG) or high glucose with metformin addition (HG + MET) following a 14-day osteogenic induction. The color key (from purple to red) of Z-score value (− 1.95 to 2.77) indicated low to high expression levels. **C** Heatmap showing the osteogenesis-related genes in PDLSCs when they were cultured in normal (CON), high glucose (HG) or high glucose with metformin addition (HG + MET) following a 14-day osteogenic induction. The color key (from purple to red) of Z-score value (− 1.24 to 2.08) indicated low to high expression levels. **D** The statistical analysis of expressed levels of osteogenesis-related genes (*AREG*, *IGFBP5*, *GPR6*8, *SFRP2* and *NPR3*) in PDLSCs when they were cultured in normal, high glucose or high glucose with metformin addition following a 14-day osteogenic induction (mRNA expression levels detected by qRT-PCR). **E** Protein expression of SFRP2 and NPR3 in PDLSCs when they were cultured in normal, high glucose or high glucose with metformin addition following a 14-day osteogenic induction (protein expression levels detected by Western blot analysis). The displayed bands were cropped from the corresponding original blots. HG, high glucose. Met, metformin. Experiments for P4 cells from three different donors were repeated independently for at least 3 times, and data are presented as the means ± SD (*n* = 3). *p *value was based on one-way analysis of variance (one-way ANOVA). *p < 0.05, **p < 0.01, and ***p < 0.001 represent significant differences between the indicated columns, while *NS* represents no significant difference

To explore potential genes involved in the metformin-enhanced osteogenic differentiation under high glucose, RNA-seq analysis was performed to analyze differentially expressed osteogenesis-related genes in PDLSCs cultured in normal (CON group), high glucose (HG group) or high glucose medium with metformin addition (HG + MET group). The threshold criteria for screening up- or downregulated genes was fold change ≥ 2.0 and *p* value ≤ 0.05. According to the results of RNA-seq analysis, 84 differentially expressed genes were screened out. Among these differentially expressed genes, 38 genes were highly expressed in HG group, and 46 genes were highly expressed in CON group and HG + MET group (Fig. 3A, B). As we were specifically interested in osteogenic differentiation, these 84 genes were further examined to identify those coding osteogenesis-related proteins, and we found that five of these 84 genes potentially contribute to cellular osteogenic differentiation. Among these five genes, *AREG*, *IGFBP5*, *GPR68* and *SFRP2* were upregulated in CON group and HG + MET group but downregulated in HG group, while *NPR3* was upregulated in HG group but downregulated in the other two groups (Fig. 3C).

To further verify these results, we examined the expression of the five genes with qRT-PCR and found that the expression patterns of *NPR3* and *SFRP2* were consistent with the results of RNA-seq analysis (Fig. 3D). We then performed Western blot analysis to validate the protein expression levels of NPR3 and SFRP2, and the results showed that the protein expression pattern of NPR3 was in line with the results of RNA-seq analysis and qRT-PCR (Fig. 3E).

### Upregulation of NPR3 compromised the metformin-enhanced osteogenic differentiation of PDLSCs under high glucose

**Fig. 4:**
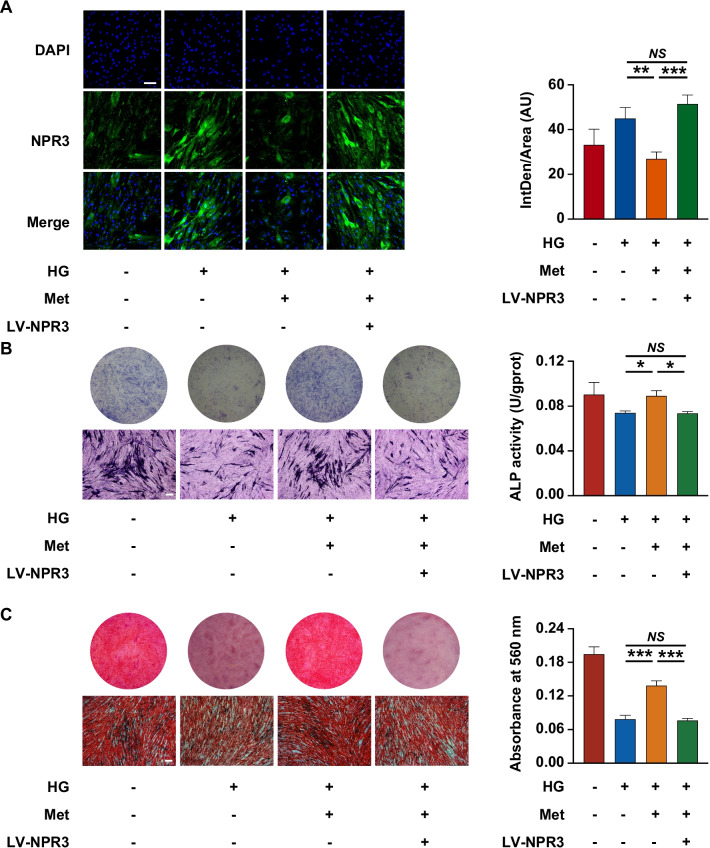
Upregulation of NPR3 compromised the metformin-enhanced osteogenic differentiation of PDLSCs under high glucose. **A** Representative confocal images and quantitative analysis of natriuretic peptide receptor 3 (NPR3) in PDLSCs transfected with or without LV-NPR3 when they were cultured in normal, high glucose, or high glucose with metformin addition following a 14-day osteogenic induction (scale bar = 100 µm, ×200 magnification). **B** ALP staining and ALP activity assay of PDLSCs transfected with or without LV-NPR3 when they were cultured in normal, high glucose, or high glucose with metformin addition following a 7-day osteogenic induction (scale bar = 200 µm). **C** Alizarin Red staining and quantitative analysis of the stained calcium deposits formed by PDLSCs transfected with or without LV-NPR3 when they were cultured in normal, high glucose, or high glucose with metformin addition following a 21-day osteogenic induction (scale bar = 200 µm). HG, high glucose. Met, metformin. LV-NPR3, NPR3-overexpression lentivirus. Experiments for P4 cells from three different donors were repeated independently for at least 3 times, and data are presented as the means ± SD (*n* = 3). *p *value was based on one-way analysis of variance (one-way ANOVA). *p < 0.05, **p < 0.01, and ***p < 0.001 represent significant differences between the indicated columns, while *NS* represents no significant difference

**Fig. 5:**
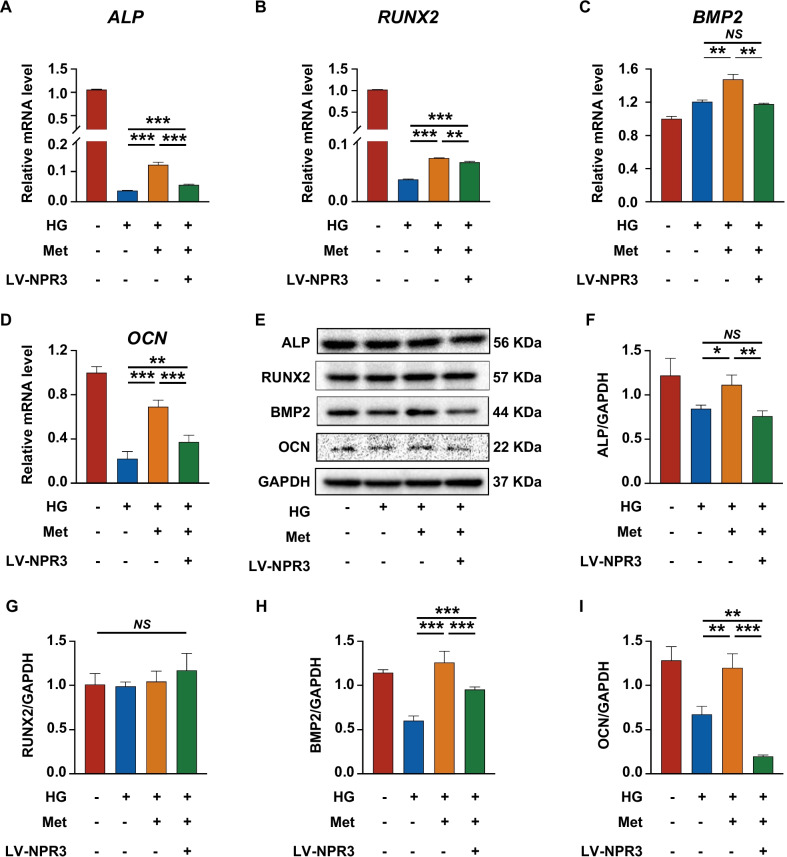
Upregulation of NPR3 decreased the metformin-enhanced expression of osteoblast differentiation-related genes and proteins. **A**–**D** Expression of osteoblast differentiation-related genes (*ALP*, *RUNX2*, *BMP2* and *OCN*) in PDLSCs transfected with or without LV-NPR3 when they were cultured in normal, high glucose, or high glucose with metformin addition following a 14-day osteogenic induction (mRNA expression levels detected by qRT-PCR). **E**–**I** Expression of osteoblast differentiation-related proteins (ALP, RUNX2, BMP2 and OCN) in PDLSCs transfected with or without LV-NPR3 when they were cultured in normal, high glucose, or high glucose with metformin addition following a 14-day osteogenic induction (protein expression levels detected by Western blot analysis). The displayed bands were cropped from the corresponding original blots. HG, high glucose. Met, metformin. LV-NPR3, NPR3-overexpression lentivirus. Experiments for P4 cells from three different donors were repeated independently for at least 3 times, and data are presented as the means ± SD (*n* = 3). *p *value was based on one-way analysis of variance (one-way ANOVA). *p < 0.05, **p < 0.01, and ***p < 0.001 represent significant differences between the indicated columns, while *NS* represents no significant difference

To further investigate the relationship between the metformin-enhanced osteogenic differentiation and the expression of NPR3, we constructed NPR3-overexpression lentivirus to stably upregulate NPR3 expression in PDLSCs. The transfection efficiency was confirmed using phase contrast and fluorescence microscopy according to the manufacturer’s instructions, as > 70% PDLSCs can express the green fluorescent protein after lentivirus transfection (Additional file 3: Fig. S3A). And the efficiency of lentivirus-mediated NPR3 upregulation was confirmed by qRT-PCR, Western blot analysis and immunofluorescence staining, as a significant increase in gene and protein expression of NPR3 after lentivirus transfection (Additional file 3: Fig. S3B–D). The expression of NPR3 in PDLSCs among the groups was visualized by immunofluorescence staining (Fig. 4A). After NPR3 upregulation, fewer ALP-positive cells and lower cellular ALP activity were found in metformin-treated PDLSCs (Fig. 4B). The results of Alizarin red staining and quantitative analysis further revealed that less calcium deposits were formed by metformin-treated PDLSCs after NPR3 upregulation (Fig. 4C). In addition, we found that metformin-enhanced gene expression of *ALP*, *RUNX2*, *BMP2* and *OCN* was reduced upon upregulation in NPR3 expression (Fig. 5A–D). The results of Western blot analysis also confirmed that the protein levels of ALP, BMP2 and OCN in metformin-treated PDLSCs were decreased after NPR3 upregulation, and no significant difference was found in the protein level of RUNX2 among the groups (Fig. 5E–I).

### Metformin-enhanced PDLSC osteogenic differentiation under high glucose via inhibition of the NPR3-mediated MAPK pathway

**Fig. 6:**
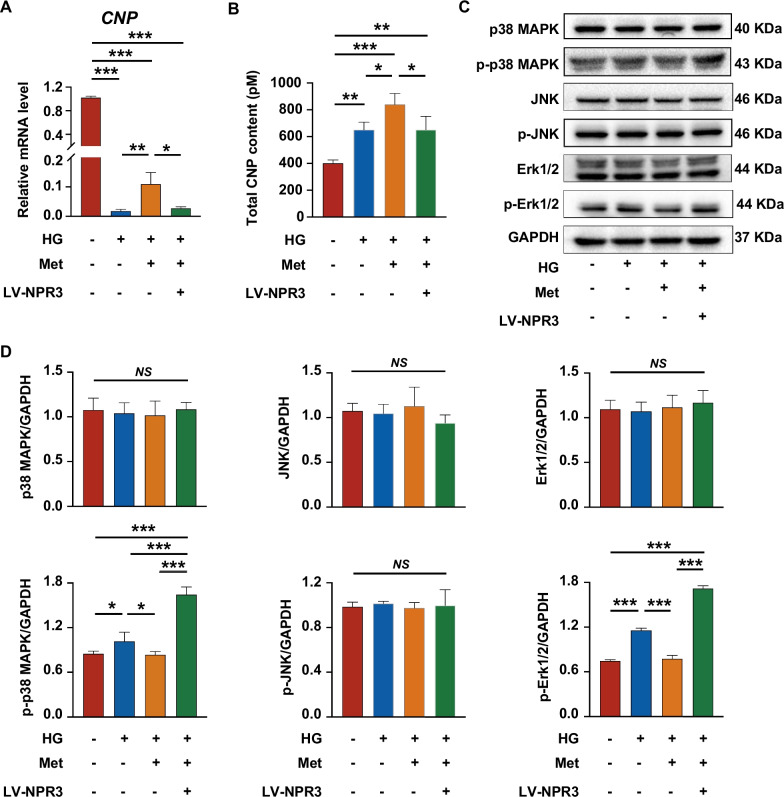
Metformin enhanced PDLSC osteogenic differentiation under high glucose via inhibition of the NPR3-mediated MAPK pathway. **A** Gene expression of C-type natriuretic peptide (CNP) in PDLSCs transfected with or without LV-NPR3 when they were cultured in normal, high glucose, or high glucose with metformin addition (mRNA expression levels detected by qRT-PCR). **B** Total CNP content in PDLSCs transfected with or without LV-NPR3 when they were cultured in normal, high glucose, or high glucose with metformin addition (total content in the cell culture supernates detected by ELISA). **C**, **D** Protein expression of MAPK pathway-related proteins (p38 MAPK, p-p38 MAPK, Erk1/2, p-Erk1/2, JNK, p-JNK) in PDLSCs transfected with or without LV-NPR3 when they were cultured in normal, high glucose, or high glucose with metformin addition (protein expression levels detected by Western blot analysis). The displayed bands were cropped from the corresponding original blots. HG, high glucose. Met, metformin. LV-NPR3, NPR3-overexpression lentivirus. Experiments for P4 cells from three different donors were repeated independently for at least 3 times, and data are presented as the means ± SD (*n* = 3). *p *value was based on one-way analysis of variance (one-way ANOVA). *p < 0.05, **p < 0.01, and ***p < 0.001 represent significant differences between the indicated columns, while *NS* represents no significant difference

To explore whether the metformin-enhanced osteogenic differentiation occurs through regulation of NPR3-mediated MAPK pathway, we detected the protein levels of critical members of MAPK pathway. We first detected the expression of C-type natriuretic peptide (CNP). The results of qRT-PCR and ELISA showed that the expression of CNP was increased in PDLSCs treated with metformin, but this increase was drastically reduced after upregulation of NPR3 (Fig. 6A, B). The results of Western blot analysis further revealed that the expression of phosphorylated p38 MAPK and Erk1/2 was upregulated in PDLSCs under high glucose and downregulated after metformin addition. However, the metformin-mediated downregulation was further reversed by upregulation of NPR3. In addition, no significant difference was observed in phosphorylated JNK and total levels of p38 MAPK, Erk1/2 and JNK protein (Fig. 6C, D).

**Fig. 7:**
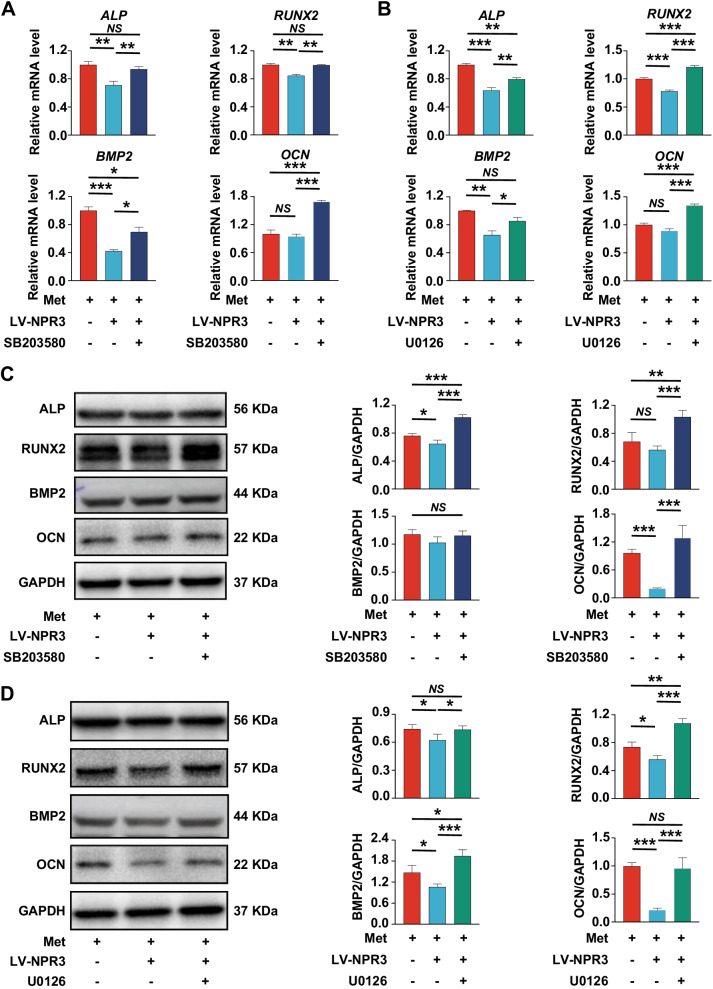
Inhibition of the NPR3-mediated p38 MAPK or Erk1/2 pathway enhanced PDLSC osteogenic differentiation under glucose. **A** Expression of osteoblast differentiation-related genes (*ALP*, *RUNX2*, *BMP2* and *OCN*) in PDLSCs transfected with or without LV-NPR3 when they were cultured in high glucose with metformin addition or high glucose with metformin and SB203580 addition following a 14-day osteogenic induction (mRNA expression levels detected by qRT-PCR). **B** Expression of osteoblast differentiation-related genes (*ALP*, *RUNX2*, *BMP2* and *OCN*) in PDLSCs transfected with or without LV-NPR3 when they were cultured in high glucose with metformin addition or high glucose with metformin and U0126 addition following a 14-day osteogenic induction (mRNA expression levels detected by qRT-PCR). **C** Expression of osteoblast differentiation-related proteins (ALP, RUNX2, BMP2 and OCN) in PDLSCs transfected with or without LV-NPR3 when they were cultured in high glucose with metformin addition or high glucose with metformin and SB203580 addition following a 14-day osteogenic induction (protein expression levels detected by Western blot analysis). **D** Expression of osteoblast differentiation-related proteins (ALP, RUNX2, BMP2 and OCN) in PDLSCs transfected with or without LV-NPR3 when they were cultured in high glucose with metformin addition or high glucose with metformin and U0126 addition following a 14-day osteogenic induction (protein expression levels detected by Western blot analysis). The displayed bands were cropped from the corresponding original blots. HG, high glucose. Met, metformin. LV-NPR3, NPR3-overexpression lentivirus. SB203580, p38 MAPK pathway inhibitor. U0126, Erk1/2 pathway inhibitor. Experiments for P4 cells from three different donors were repeated independently for at least 3 times, and data are presented as the means ± SD (*n* = 3). *p *value was based on one-way analysis of variance (one-way ANOVA). *p < 0.05, **p < 0.01, and ***p < 0.001 represent significant differences between the indicated columns, while *NS* represents no significant difference

To further validate our findings, p38 MAPK and Erk1/2 pathway were inhibited with specific inhibitors SB203580 (MedChemExpress, Monmouth Junction, NJ, USA) and U0126 (MedChemExpress) respectively. The results of qRT-PCR showed that the gene levels of *ALP*, *RUNX2*, *BMP2* and *OCN* were significantly increased after inhibition of p38 MAPK (Fig. 7A) or Erk1/2 pathway (Fig. 7B). Similarly, the results of Western blot analysis revealed that the protein levels of ALP, RUNX2 and OCN in NPR3-upregulated PDLSCs were also increased after inhibition of p38 MAPK (Fig. 7C) or Erk1/2 pathway (Fig. 7D). But no obvious change was found in the protein level of BMP2 after inhibition of p38 MAPK pathway (Fig. 7C).

## Discussion

Periodontal disease, a leading cause of tooth loss in adults, can cause progressive destruction of the tooth-supporting apparatus [1]. In the past decades, MSCs, particularly PDLSCs, have become promising candidates for repairing alveolar bone defect and mediating periodontal tissue regeneration [12, 15]. However, accumulating evidence suggests that long-term exposure to diabetic conditions might cause cell damage; and such damage has long been a challenge to the therapeutic effect of stem cells in diabetic individuals [31, 32]. In addition, the development of a chronic hyperglycemic environment may be responsible for a range of diabetic complications and cell dysfunction [4]. Previous studies have revealed the damage of high glucose on MSCs, including weakened mobilization and proliferation capacity, impaired differentiation potential and immunoregulatory capacity [16, 31, 33]. Thus, we first chose 25 mM glucose to mimic a high glucose condition according to previous studies and further explored the effects of high glucose on the osteogenic differentiation of PDLSCs [33–36]. Similarly, we found that incubation of PDLSCs under high glucose led to impaired osteogenic differentiation, evidenced by a decrease in formation of calcium deposit and expression of osteoblast differentiation-related genes and proteins. Therefore, our findings verify that long-term incubation in high glucose can reduce the stem cell performance of PDLSCs, as was found in previous studies, and further indicate the need to protect cells from high glucose-induced damage.

Metformin is a potent insulin-sensitizing drug for the treatment of diabetes mellitus [37]. In addition to its anti-hyperglycemic effect, metformin has been found to exert protective effects on different cell types [38–40]. Moreover, metformin acts in a concentration-dependent manner; and the concentrations of metformin used for the treatment of MSCs typically range from 10 to 1000 µM [38, 41–43]. Therefore, we first selected a feasible concentration of metformin using CCK-8 assay. We found that metformin at concentrations ranging from 0 to 1000 µM induced no cytotoxicity and that metformin at the concentration of 100 µM conferred the maximal protective effect on cell viability under high glucose. Furthermore, we added 100 µM metformin into the culture medium. The results of qRT-PCR and Western blot analysis showed that the expression of osteoblast differentiation-related genes and proteins in PDLSCs under high glucose was increased after addition of metformin, suggesting that metformin can enhance the osteogenic differentiation ability of PDLSCs under high glucose. Similarly, previous studies have reported that 100 µM metformin can improve the differentiation potential of cells from aged rats [38]. In addition, metformin at concentrations ranging from 10 to 100 µM can enhance the migration potential of PDLSCs; several studies have also revealed that 1 mM metformin treatment can protect MSCs from apoptosis under inflammatory conditions [24, 42]. The different metformin concentrations may be due to the distinct culture conditions, which could alter the cell sensitivity to metformin. Moreover, it has been reported that, after oral administration (35 mg/kg body weight), the plasma concentrations of metformin in the portal vein are 40–70 µM [44]. Though the metformin concentration in periodontal tissue has not been clear, some studies suggested that metformin at concentrations of 70–120 µM, higher than the plasma concentrations in the portal vein, can be used for the treatment of periodontitis [45, 46]. These studies indicated that the low metformin concentration in periodontal tissues after oral administration may be responsible for the failure of the treatment dose of metformin to improve periodontal tissue regeneration. In addition, though previous studies have demonstrated that high glucose leads to decreased expression of RUNX2, we found in this study that the protein level of RUNX2 was not changed under high glucose or following metformin treatment [33, 47]. It has been reported that the expression of this transcription factor is increased at the early stage of osteogenic differentiation but is decreased at the late stage [48]. In this part of the experiments, we detected the protein expression of RUNX2 at 14 days after osteogenic induction, the late stage of osteogenic differentiation. These might explain why the protein level of RUNX2 was not obviously altered in the PDLSCs under high glucose or following metformin treatment. Taken together, the present and previous results suggest that metformin may exert a protective effect on the treatment of stem cells, including (but not limited to) PDLSCs, and the effects of metformin are not only dependent on the drug concentration, but are also different in diverse cell lineages and under different incubation conditions. Moreover, our findings suggest that metformin at the concentration of 100 µM may be feasible for treatment of PDLSCs under high glucose.

To further explore the underlying mechanisms, we conducted RNA-seq analysis to investigate the potential genes mediating the effect of metformin on PDLSCs under high glucose. Five osteogenesis-related genes *AREG*, *IGFBP5*, *GPR68*, *SFRP2* and *NPR3* were detected. Among these genes, *AREG*, *IGFBP5*, *GPR68* and *SFRP2* were downregulated in PDLSCs under high glucose and upregulated after the addition of metformin, while *NPR3* exhibited the opposite pattern. We further confirmed the mRNA and protein expression levels of these five genes and found that only the mRNA and protein expression of NPR3 was consistent with the RNA-seq results. Thus, NPR3 was considered as a potential target of metformin in further experiments. To further investigate the role of NPR3 in metformin-enhanced osteogenic differentiation under high glucose, we used NPR3-overexpression lentivirus to stably upregulate NPR3 expression in PDLSCs. After upregulation of NPR3, we found that the calcium deposit formation, the expression of osteoblast differentiation-related genes and proteins were decreased in metformin-treated PDLSCs. These data suggest upregulation of NPR3 can compromise the metformin-enhanced PDLSC osteogenic differentiation. NPR3 is a clearance receptor of natriuretic peptides and has been reported to be involved in regulation of cell metabolism and progression of metabolic diseases [49, 50]. Consistent with our findings, several studies suggest that NPR3 expression is upregulated in adipose tissues from diabetic individuals [51]; and inhibition of NPR3 can stimulate endochondral ossification and bone growth [52, 53]. Therefore, our results suggest that high glucose-induced damage to PDLSCs might be linked to upregulation of NPR3 and that metformin can enhance the osteogenic differentiation of PDLSCs under high glucose via downregulation of NPR3. Moreover, regulation of NPR3 may be an effective strategy for maintaining the functionality of cells under high glucose.

Mitogen-activated protein kinase (MAPK) pathway is a downstream signaling pathway of NPR3. The intracellular Gi/o-binding domain of NPR3 could increase the phosphorylation level of ERK1/2 and further activate the MAPK pathway [49, 54]. In addition, NPR3 can also increase the level of phosphorylated ERK1/2 through decreasing bioavailable CNP [55]. Thus, we further detected the changes in the three major subfamilies of MAPK pathway, p38 MAPK, JNK and Erk1/2 pathway. After upregulation of NPR3, the metformin-mediated decrease in phosphorylation of p38 MAPK and Erk1/2 was reversed, but not JNK. These results suggest that upregulation of NPR3 can activate the MAPK pathway (particularly the p38 MAPK and Erk1/2 pathway) in PDLSCs treated with metformin, indicating that the NPR3-mediated MAPK pathway may be involved in the metformin-enhanced PDLSC osteogenic differentiation.

MAPK pathway plays a crucial role in cellular differentiation. Gold nanoparticles can enhance osteogenic differentiation of MSCs by the p38 MAPK pathway; and osteoblasts lacking p38 MAPK pathway-related proteins show decreased marker gene expression and defective mineralization [56, 57]. In addition, Erk1/2 pathway has also been suggested to be one of the key regulators in the osteogenic differentiation of PDLSCs [58, 59]. To further validate the role of NPR3-mediated MAPK pathway in the metformin-enhanced PDLSC osteogenic differentiation, we blunted the p38 MAPK and Erk1/2 pathway with the corresponding inhibitors (the phosphorylation-specific inhibitor for p38 MAPK was SB203580 while that for Erk1/2 was U0126) and observed the corresponding effects on PDLSC osteogenic differentiation. To block the p38 MAPK and Erk1/2 pathway, the concentration (20 μM) and duration (2 h) of SB203580 and U0126 on PDLSCs were selected according to similar investigations [59, 60]. From the results of qRT-PCR and Western blot analysis, we identified that the osteogenic differentiation of NPR3-upregulated PDLSCs was enhanced after the addition of p38 MAPK or Erk1/2 inhibitors, suggesting that the p38 MAPK and Erk1/2 pathways are closely related to the function of NPR3, which is consistent with the above speculation. However, although previous studies have also demonstrated that the JNK pathway is crucial for the osteogenic differentiation of MSCs, we found no obvious change in the JNK pathway after NPR3 upregulation, indicating that the JNK pathway might not be a target of NPR3 [61]. Taken together, our results suggest that metformin might enhance PDLSC osteogenic differentiation under high glucose via downregulation of NPR3 and inhibition of the downstream MAPK pathway.

**Fig. 8:**
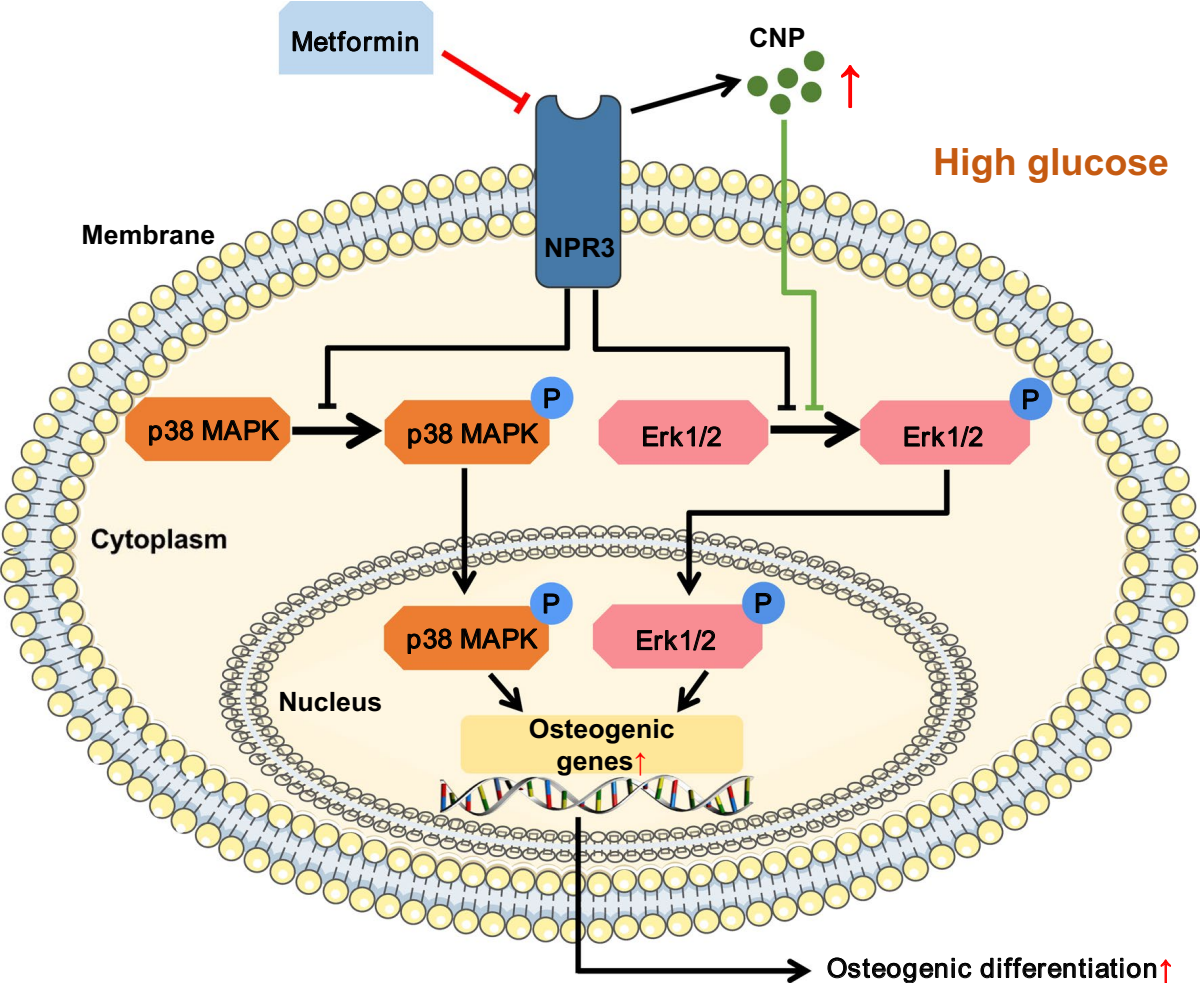
Schematic of the identified signaling molecules/pathways involved in the metformin-enhanced osteogenic differentiation of periodontal ligament stem cells under high glucose

In summary, our study revealed for the first time that metformin can enhance the osteogenic differentiation of PDLSCs under high glucose and that metformin functions via downregulating NPR3 and inhibiting the downstream MAPK pathway (Fig. 8). These results indicated that NPR3-mediated MAPK pathway inhibitors, including but not limited to metformin, may be used as therapeutic agents to promote stemness of stem cells like PDLSCs and enhance their therapeutic efficiency in cellular therapy in the future. In addition, the limitations of our study should be acknowledged. We only used an in *vitro* model in this study to investigate the effect of metformin on PDLSCs under high glucose. Further in *vivo* studies are needed to fully elucidate the intracellular responses underlying the effect of metformin under diabetic conditions.

## Conclusions

In this study, we prove for the first time that the function of metformin in combating high glucose-damaged osteogenic differentiation was related to its role as a regulator of NPR3. Further evidence suggests that MAPK pathway, particularly the p38 MAPK and Erk1/2 pathway, can be activated by NPR3, and the NPR3-mediated MAPK pathway was involved in the metformin-enhanced osteogenic differentiation under high glucose. This is the first report to demonstrate that metformin-enhanced osteogenic differentiation under high glucose occurs via downregulating NPR3 and inhibiting the downstream MAPK pathway, indicating that regulation of the NPR3-mediated MAPK pathway can be considered as a key therapeutic target for effective periodontal regeneration in diabetic individuals.

## Supplementary Information

**Additional file 1: Fig. S1.** Isolation and characterization of PDLSCs. **A** Colony formation ability of PDLSCs: colonies in a macroscopic view and a single colony observed by microscopy (scale bar = 200 µm). **B** Proliferative activity of PDLSCs assessed by CCK-8 assay during an 8-day culture (*n* = 3). **C** Alizarin red staining (left; scale bar = 200 µm), Oil red O staining (middle; scale bar = 200 µm) and Alcian blue staining (right; scale bar = 200 µm) of the PDLSCs following a 21-day osteogenic, adipogenic or chondrogenic induction. **D** Surface markers of PDLSCs assessed by flow cytometry analysis.

**Additional file 2: Fig. S2.** Selection of the optimal concentration of metformin. **A** Cell viability of PDLSCs during a 72-h metformin treatment at the indicated concentrations (cell viability detected by CCK-8 assay). **B** Cell viability of PDLSCs during a 6-day metformin treatment at the indicated concentrations (cell viability detected by CCK-8 assay). M0, high glucose without metformin addition. M1, high glucose with 10 µM metformin addition. M2, high glucose with 100 µM metformin addition. M3, high glucose with 500 µM metformin addition. M4, high glucose with 1000 µM metformin addition. Experiments for P4 cells from three different donors were repeated independently for at least 3 times and data are presented as the means ± SD (*n* = 3). *p *value was based on two-way analysis of variance (two-way ANOVA). ^*p* < 0.05, ^^*p* < 0.01 and ^^^*p* < 0.001 represent significant differences between M2 and M1; #*p* < 0.05, ##*p* < 0.01 and ###*p* < 0.001 represent significant differences between M2 and M3; $*p* < 0.05, $$*p* < 0.01 and $$$*p* < 0.001 represent significant differences between M2 and M4, while *NS* represents no significant difference.

**Additional file 3: Fig. S3.** The efficiency of lentivirus-mediated upregulation of NPR3 expression. **A** GFP expression in PDLSCs after transfection with lentiviral vector for 72 h (scale bar = 100 µm, × 100 magnification). **B** Gene expression of NPR3 in PDLSCs in response to transfection with LV-NC or LV-NPR3 (mRNA expression levels detected by qRT-PCR). **C** Protein expression of NPR3 in PDLSCs in response to transfection with LV-NC or LV-NPR3 for 24 h or 72 h (protein expression levels detected by Western blot analysis). **D** Representative confocal images of natriuretic peptide receptor 3 (NPR3) in PDLSCs in response to transfection with LV-NC or LV-NPR3 (scale bar = 100 µm, ×200 magnification). GFP, green fluorescent protein. LV-NC, LV5 lentiviral vector. LV-NPR3, NPR3-overexpression lentivirus. Experiments for P4 cells from three different donors were repeated independently for at least 3 times and data are presented as the means ± SD (*n* = 3). *p *value was based on *t* test. *p < 0.05, **p < 0.01, and ***p < 0.001 represent significant differences between the indicated columns, while *NS* represents no significant difference.

## Data Availability

All data generated or analyzed during this study are included in this published article.

## Abbreviations

MSCs: Mesenchymal stem cells
PDL: Periodontal ligament
PDLSCs: Periodontal ligament stem cells
CFU: Colony-forming unit
CCK-8: Cell Counting Kit-8
ALP: Alkaline phosphatase
NPR3: Natriuretic peptide receptor 3
ELISA: Enzyme-linked immunosorbent assay
CNP: C-type natriuretic peptide
qRT-PCR: Quantitative real-time polymerase chain reaction
MAPK: Mitogen-activated protein kinase
