# Supplementary figures and images for: Metformin combats high glucose-induced damage to the osteogenic differentiation of human periodontal ligament stem cells via inhibition of the NPR3-mediated MAPK pathway
Source: Stem Cell Res Ther. 2022 Jul 15;13:305. doi: 10.1186/s13287-022-02992-z (PMC9284897; doi:10.1186/s13287-022-02992-z)

**A****CFU**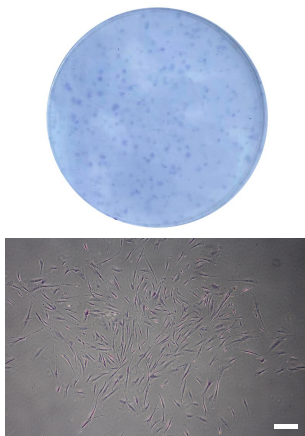**B**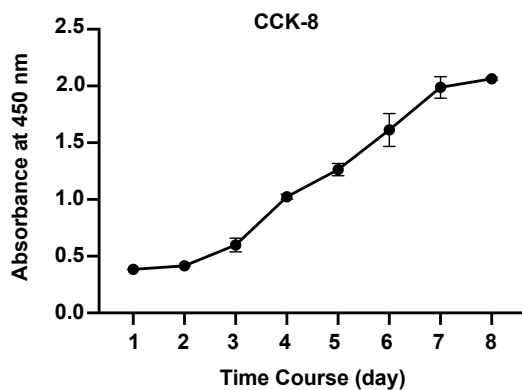**C****Alizarin Red staining**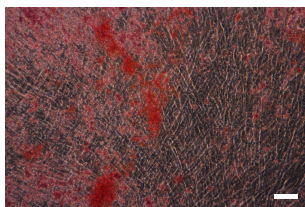**Oil Red O staining**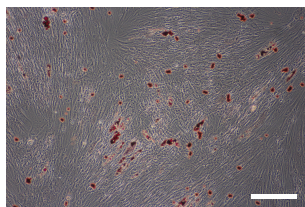**Alcian blue staining**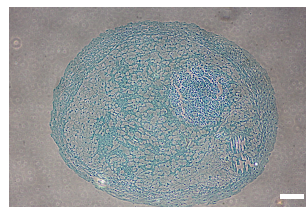**D**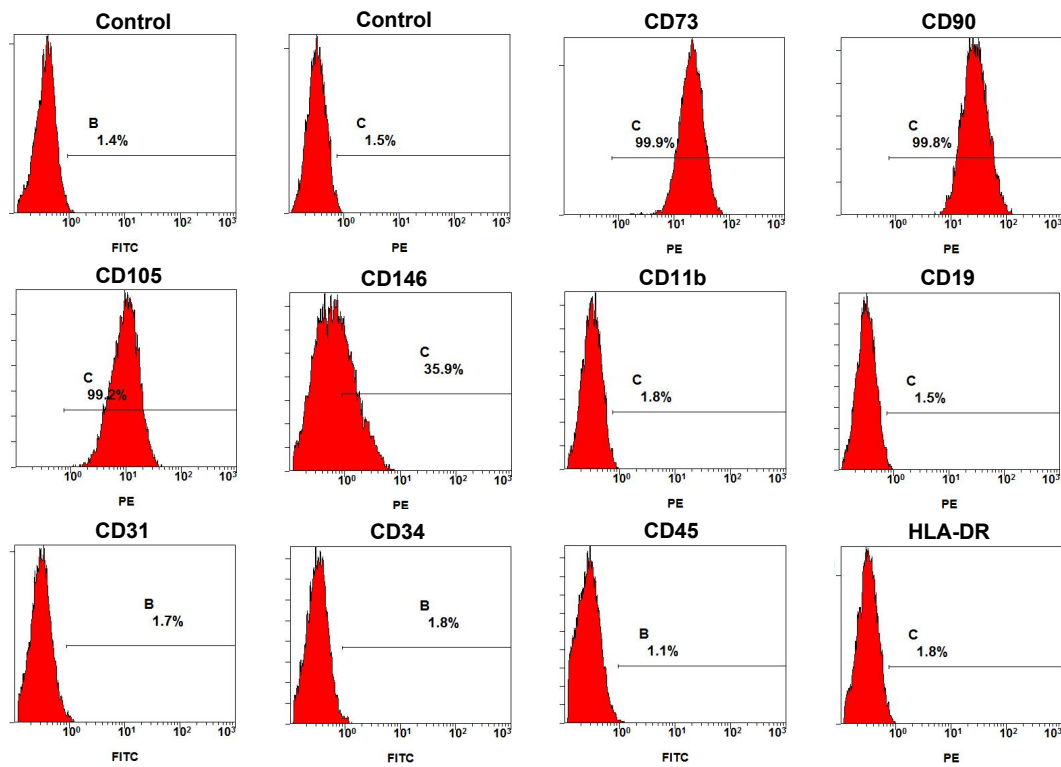

Supplement: Supplementary file 1 — Additional file 1: Fig. S1. Isolation and characterization of PDLSCs. A Colony formation ability of PDLSCs: colonies in a macroscopic view and a single colony observed by microscopy (scale bar = 200 µm). B Proliferative activity of PDLSCs assessed by CCK-8 assay during an 8-day culture (n = 3). C Alizarin red staining (left; scale bar = 200 µm), Oil red O staining (middle; scale bar = 200 µm) and Alcian blue staining (right; scale bar = 200 µm) of the PDLSCs following a 21-day osteogenic, adipogenic or chondrogenic induction. D Surface markers of PDLSCs assessed by flow cytometry analysis. [file 13287_2022_2992_MOESM1_ESM.pdf]

**A**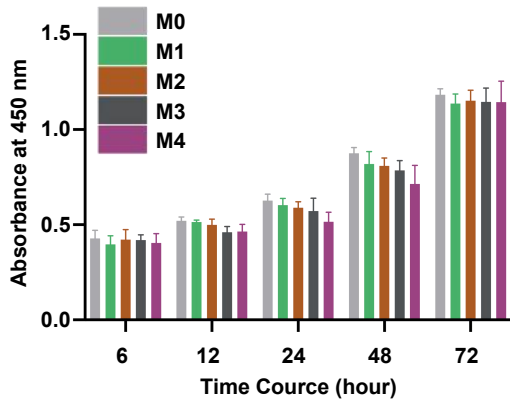**B**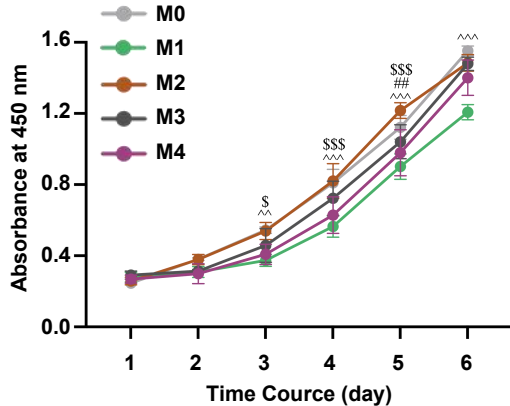

Supplement: Supplementary file 2 — Additional file 2: Fig. S2. Selection of the optimal concentration of metformin. A Cell viability of PDLSCs during a 72-h metformin treatment at the indicated concentrations (cell viability detected by CCK-8 assay). B Cell viability of PDLSCs during a 6-day metformin treatment at the indicated concentrations (cell viability detected by CCK-8 assay). M0, high glucose without metformin addition. M1, high glucose with 10 µM metformin addition. M2, high glucose with 100 µM metformin addition. M3, high glucose with 500 µM metformin addition. M4, high glucose with 1000 µM metformin addition. Experiments for P4 cells from three different donors were repeated independently for at least 3 times and data are presented as the means ± SD (n = 3). p value was based on two-way analysis of variance (two-way ANOVA). ^p < 0.05, ^^p < 0.01 and ^^^p < 0.001 represent significant differences between M2 and M1; #p < 0.05, ##p < 0.01 and ###p < 0.001 represent significant differences between M2 and M3; $p < 0.05, $$p < 0.01 and $$$p < 0.001 represent significant differences between M2 and M4, while NS represents no significant difference. [file 13287_2022_2992_MOESM2_ESM.pdf]

**A**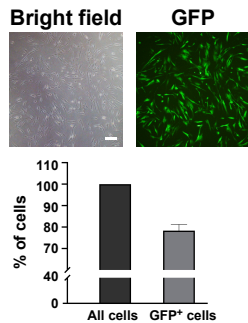**B**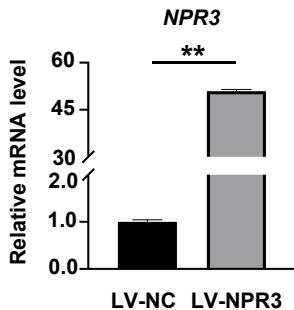**C**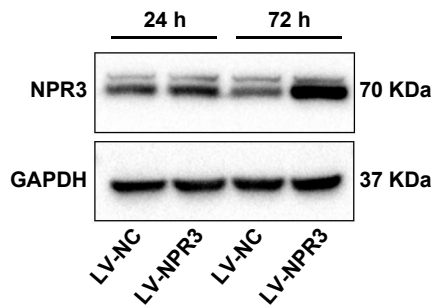**D**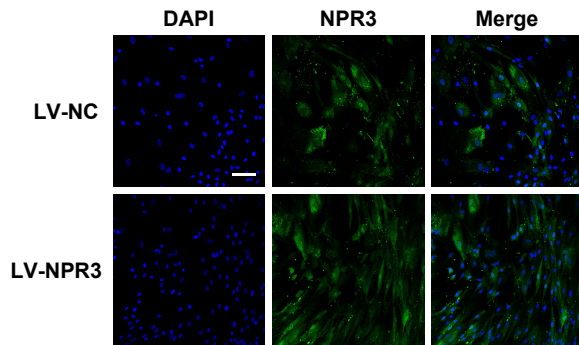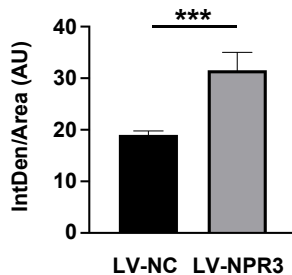

Supplement: Supplementary file 3 — Additional file 3: Fig. S3. The efficiency of lentivirus-mediated upregulation of NPR3 expression. A GFP expression in PDLSCs after transfection with lentiviral vector for 72 h (scale bar = 100 µm, × 100 magnification). B Gene expression of NPR3 in PDLSCs in response to transfection with LV-NC or LV-NPR3 (mRNA expression levels detected by qRT-PCR). C Protein expression of NPR3 in PDLSCs in response to transfection with LV-NC or LV-NPR3 for 24 h or 72 h (protein expression levels detected by Western blot analysis). D Representative confocal images of natriuretic peptide receptor 3 (NPR3) in PDLSCs in response to transfection with LV-NC or LV-NPR3 (scale bar = 100 µm, ×200 magnification). GFP, green fluorescent protein. LV-NC, LV5 lentiviral vector. LV-NPR3, NPR3-overexpression lentivirus. Experiments for P4 cells from three different donors were repeated independently for at least 3 times and data are presented as the means ± SD (n = 3). p value was based on t test. *p < 0.05, **p < 0.01, and ***p < 0.001 represent significant differences between the indicated columns, while NS represents no significant difference. [file 13287_2022_2992_MOESM3_ESM.pdf]
